# Identification of heterogeneous subtypes and a prognostic model for gliomas based on mitochondrial dysfunction and oxidative stress-related genes

**DOI:** 10.3389/fimmu.2023.1183475

**Published:** 2023-06-02

**Authors:** Junsheng Li, Siyu Wang, Xiaojing Chi, Qiheng He, Chuming Tao, Yaowei Ding, Jia Wang, Jizong Zhao, Wen Wang

**Affiliations:** ^1^ Department of Neurosurgery, Beijing Tiantan Hospital, Capital Medical University, Beijing, China; ^2^ China National Clinical Research Center for Neurological Diseases, Beijing, China; ^3^ Center of Stroke, Beijing Institute for Brain Disorders, Beijing, China; ^4^ Beijing Key Laboratory of Translational Medicine for Cerebrovascular Disease, Beijing, China; ^5^ Beijing Translational Engineering Center for 3D Printer in Clinical Neuroscience, Beijing, China; ^6^ NHC Key Laboratory of Systems Biology of Pathogens, Institute of Pathogen Biology, Chinese Academy of Medical Sciences & Peking Union Medical College, Beijing, China; ^7^ Department of Neurosurgery, Second Affiliated Hospital of Soochow University, Suzhou, China; ^8^ Department of Clinical Diagnosis, Laboratory of Beijing Tiantan Hospital, Capital Medical University, Beijing, China; ^9^ Savaid Medical School, University of the Chinese Academy of Sciences, Beijing, China

**Keywords:** mitochondrial dysfunction, oxidative stress, glioma, molecular subtype, prognosis

## Abstract

**Objective:**

Mitochondrial dysfunction and oxidative stress are known to involved in tumor occurrence and progression. This study aimed to explore the molecular subtypes of lower-grade gliomas (LGGs) based on oxidative stress-related and mitochondrial-related genes (OMRGs) and construct a prognostic model for predicting prognosis and therapeutic response in LGG patients.

**Methods:**

A total of 223 OMRGs were identified by the overlap of oxidative stress-related genes (ORGs) and mitochondrial-related genes (MRGs). Using consensus clustering analysis, we identified molecular subtypes of LGG samples from TCGA database and confirmed the differentially expressed genes (DEGs) between clusters. We constructed a risk score model using LASSO regression and analyzed the immune-related profiles and drug sensitivity of different risk groups. The prognostic role of the risk score was confirmed using Cox regression and Kaplan-Meier curves, and a nomogram model was constructed to predict OS rates. We validated the prognostic role of OMRG-related risk score in three external datasets. Quantitative real-time PCR (qRT-PCR) and immunohistochemistry (IHC) staining confirmed the expression of selected genes. Furthermore, wound healing and transwell assays were performed to confirm the gene function in glioma.

**Results:**

We identified two OMRG-related clusters and cluster 1 was significantly associated with poor outcomes (P<0.001). The mutant frequencies of IDH were significantly lower in cluster 1 (P<0.05). We found that the OMRG-related risk scores were significantly correlated to the levels of immune infiltration and immune checkpoint expression. High-risk samples were more sensitive to most chemotherapeutic agents. We identified the prognostic role of OMRG-related risk score in LGG patients (HR=2.665, 95%CI=1.626-4.369, P<0.001) and observed that patients with high-risk scores were significantly associated with poor prognosis (P<0.001). We validated our findings in three external datasets. The results of qRT-PCR and IHC staining verified the expression levels of the selected genes. The functional experiments showed a significant decrease in the migration of glioma after knockdown of SCNN1B.

**Conclusion:**

We identified two molecular subtypes and constructed a prognostic model, which provided a novel insight into the potential biological function and prognostic significance of mitochondrial dysfunction and oxidative stress in LGG. Our study might help in the development of more precise treatments for gliomas.

## Introduction

Glioma is a type of neoplastic disease that originates from glial cells, and it is the most common intracranial malignancy (1). According to the WHO classification, gliomas are classified as lower-grade glioma (grade II/III, LGG) and glioblastoma (grade IV, GBM) (2, 3). LGG is typically a slow-growing indolent precursor compared to GBM (4). However, due to its invasive growth pattern, it is difficult to achieve complete tumor removal with surgical treatment, and patients are often at a high risk of local recurrence and malignant progression into secondary high-grade gliomas (5). Given the genetic heterogeneity in LGG, patients with similar clinical characteristics may have different overall survival (OS) rates (6). Recent advances in molecular and genetic profiling have improved our understanding of the underlying biology of gliomas and have provided new opportunities for prognostic prediction and targeted therapy. For example, mutations in the isocitrate dehydrogenase (IDH) gene are common in LGG and are associated with a more favorable prognosis (7). Additionally, other genetic alterations such as mutations in the TP53 and ATRX genes appear to be potential therapeutic targets (8, 9). Despite the advances, LGG remains a challenging clinical problem with a wide range of clinical and molecular heterogeneity. Therefore, further studies are needed to identify molecular subtypes that could contribute to the development of novel prognostic biomarkers and effective therapeutic targets.

Mitochondria are critical organelles involved in energy metabolism and cellular homeostasis. Mitochondria play a key role in providing energy for cell metabolism, differentiation, and apoptosis through oxidative phosphorylation, as well as in other cellular processes such as calcium signaling, lipid metabolism, and the production of reactive oxygen species (ROS) (10). Mitochondrial dysfunction has been shown to contribute to tumor initiation, progression, and therapy resistance by altering cellular metabolism, redox homeostasis, and signal transduction pathways. It has been well-documented that mitochondrial dysfunction could increase the ROS accumulation (11), leading to oxidative damage, which further contributes to mitochondrial dysfunction and generates more ROS (12). This creates a vicious cycle of increased oxidative stress and mitochondrial dysfunction. Excess ROS is associated with the cellular component damage, inhibition of energy metabolism, mtDNA oxidation and mutation, and genetic instability (13). Additionally, ROS can activate signaling pathways that promote tumor cell proliferation and survival, such as the MAPK and PI3K/Akt pathways (14, 15). Accumulating evidence has suggested that mitochondrial dysfunction and oxidative stress are related to the occurrence, progression, and drug resistance of tumors (16). Targeting mitochondrial dysfunction and oxidative stress is a promising therapeutic strategy for tumor treatment. Therefore, exploring molecular characteristics related to mitochondrial dysfunction and oxidative stress may aid in developing new therapeutic strategies for LGG patients.

In this study, we aimed to identify molecular subtypes in LGG patients based on oxidative stress and mitochondrial-related genes (OMRGs). We analyzed the differentially expressed genes (DEGs) between different OMRG subtypes, and constructed an OMRG-related risk score model with TCGA database. We further investigated and validated the prognostic value of the OMRG-related signature using four independent datasets. Overall, this study contributed to the classification of molecular subtype on mitochondrial dysfunction and oxidative stress, as well as the accurate prognosis in LGG patients.

## Methods

### Information collection

We collected the data of 529 LGG samples from TCGA database. A list of 1399 oxidative stress-related genes (ORGs) was obtained from Genecards database, which has been used in previous studies (17, 18). The mitochondrial-related gene (MRG) list containing a total of 1136 MRGs was obtained from the MitoCarta3.0 database (19). The overlapping genes between ORGs and MRGs were identified as oxidative stress and mitochondrial-related genes (OMRGs). The potential biological functions of OMRGs were further investigated by GO and KEGG analyses using the ClusteProfiler package (20).

### Consensus clustering

To classify samples into distinct clusters, consensus clustering has been performed by ConsensuClusterPlus package based on the OMRG expression data (21). Then 500 bootstrapping operations were performed using the km method and Canberra as the metric distance, and each bootstrap contained 80% of the samples. The clustering variable k varied from 2 to 10. We performed PCA analysis to distinguish OMRG clusters. Then mutation profiles of the samples in different clusters have been shown. Furthermore, the frequencies of the top 10 mutant genes in LGGs have been compared between clusters.

### Construction of OMRG-related prognostic model

DEGs between different clusters were confirmed by the DESeq2 package (|logFC|>2, Adjusted P<0.05) (22). The genetic interaction of DEGs has been analyzed by GeneMANIA platform (23). The DEGs with prognostic value were further included in LASSO regression via glmnet package and the lambda with minimized deviations has been selected (24). Then the filtered genes were used to establish the risk system (25).

All patients were stratified into two groups based on the median risk score. The prognostic role of the risk score was identified by Cox regression and Kaplan-Meier curves. And nomogram generated by RMS package was used to predict the OS rates. The predictive accuracy of nomogram has been evaluated by ROC curves and calibration plots.

### Gene set enrichment analysis (GSEA)

We performed GSEA to explore the functional annotations between the two groups using the ClusteProfiler package. For each analysis, the gene set permutation has been defined for 1000 times. As the filter condition, adjusted P-value was set to <0.05 and FDR value was set to <0.25.

### Immune and drug sensitivity analyses

The correlations between infiltrating immune cells and risk score were identified using single-sample GSEA (ssGSEA) function of GSVA package (26, 27). The levels of documented immune checkpoints were compared between different risk groups. The sensitivity of patients in different risk groups to chemotherapeutic agents has been compared using IC50 value by pRRophetic package (28).

### Validation of the prognostic role

The validation of the prognostic role of the OMRG-related risk score was performed in a total of 961 LGG samples from three external datasets, including 625 samples from CGGA-sequencing set, 174 samples from CGGA-microarray set, and 162 samples from REMBRANDT cohort.

### Quantitative Real-Time PCR (qRT-PCR)

Total RNA was isolated from 12 glioma tissues, including 3 grade II, 3 grade III, and 6 grade IV, and 5 paired normal tissues using TRIzol reagent (Takara, Kyoto, Japan). All tissues were collected after surgical resection and stored at −80°C until tested. The isolated RNA was reverse transcribed by PrimeScript™ RT reagent Kit (Takara, Kyoto, Japan), and qRT-PCR was performed by TB Green Premix Ex Taq (Takara, Kyoto, Japan) on the ABI Prism 7900 System. The relative quantification of signature-gene expression was performed by the 2^−ΔΔCT^ method, and GAPDH was used as the internal normalization control. The primer pairs used for qRT-PCR were listed in **Supplementary Table S1**.

### Immunohistochemistry (IHC) staining

To verify the protein levels of genes in the OMRG-related risk model between different grades of gliomas, IHC results from the Human Protein Atlas (HPA) database were used. This verified the consistency between protein level and gene expression (29).

### Cell culture and transfection

The SNB-19 cell line was obtained from the American Type Culture Collection (ATCC, USA) and cultured in DMEM (Gibco, USA) supplemented with 10% FBS (Gibco, USA), penicillin, and streptomycin. The cells were maintained in a humidified incubator at 37°C with 5% carbon dioxide and allowed to grow to confluence before transfection. For siRNA knockdown experiments, cells were transfected with siRNA kit (Ribobio, geneOFF h-SCNN1B) using Lipofectamine 3000 (Invitrogen, Carlsbad, CA) according to the manufacturer’s instructions. The control groups were transfected with siNC (negative control). The transfection efficiency was assessed by qRT-PCR.

### Wound healing assay

The wound healing assays were performed using Ibidi Culture-Insert (Ibidi, Germany). The SNB-19 cell line was suspended in complete medium at 20,000 cells/ml, and 70µl of cell suspensions were pipetted into each chamber of the cell culture insert. After 24 hours, the Culture-Insert was gently removed using sterile tweezers, and the well was filled with serum-free medium to exclude the effect of cell proliferation. Images were photographed at 24 hours after the scratch was made using an inverted-phase microscope (IX51, Olympus, Japan).

### Transwell assay

The cell migration ability was determined using transwell chamber (8μm, 24-well insert, Costar, USA). For the migration assay, cells after 24h transfection were added to the upper chamber, and medium containing 10% FBS was added to the lower chamber. Then, the migrated cells were fixed and stained with 0.1% crystal violet.

### Statistical analysis

The R project (3.6.3) has been utilized for all statistical analyses. The comparison of continuous data between different groups was conducted using Wilcoxon rank-sum and Kruskal-Wallis tests. Spearman correlation tests were used for correlation evaluation. The prognostic role of the OMRG-related risk score was evaluated by Cox regression. The survival probabilities were estimated by Kaplan-Meier curves. A two-sided P-value <0.05 was considered to be statistical significance in this study.

## Results

### Identification of OMRGs and functional enrichment analyses

**Figure 1:**
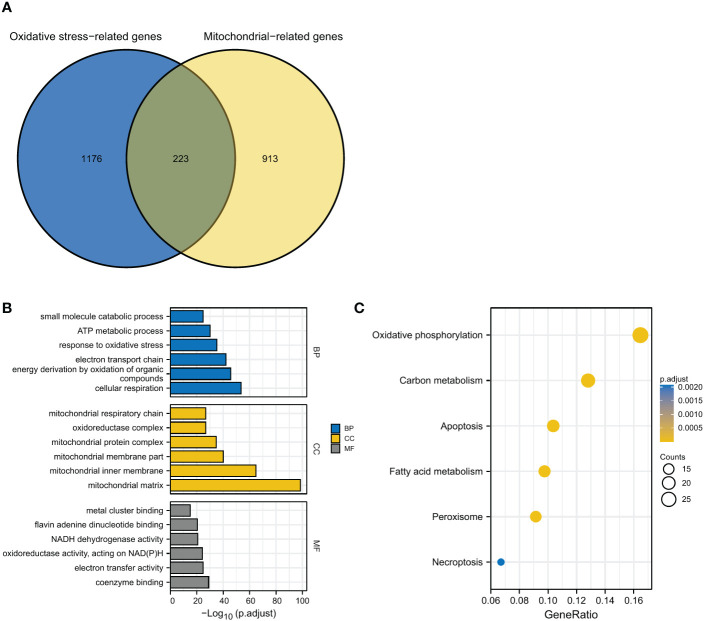
Identification and functional enrichment analyses of OMRGs. **(A)** Identification of OMRGs. **(B)** GO enrichment analyses. **(C)** KEGG analysis annotation.

After overlapping the 1399 ORGs and 1136 MRGs, a total of 223 OMRGs were identified (**Figure 1A**). The results of GO analyses have been shown (**Figure 1B**). The biological process category included cellular respiration, energy derivation by oxidation of organic compounds, electron transport chain, response to oxidative stress, ATP metabolic process, and small molecule catabolic process. The cellular component category included mitochondrial matrix, mitochondrial inner membrane, mitochondrial membrane part, mitochondrial protein complex, oxidoreductase complex, and mitochondrial respiratory chain. The molecular function category included coenzyme binding, electron transfer activity, oxidoreductase activity acting on NAD(P)H, NADH dehydrogenase activity, flavin adenine dinucleotide binding, and metal cluster binding (**Figure 1B**). KEGG analyses showed an enrichment in oxidative phosphorylation, carbon metabolism, apoptosis, fatty acid metabolism, peroxisome, and necroptosis (**Figure 1C**).

### Identification of oxidative stress and mitochondrial function-related clusters

**Figure 2:**
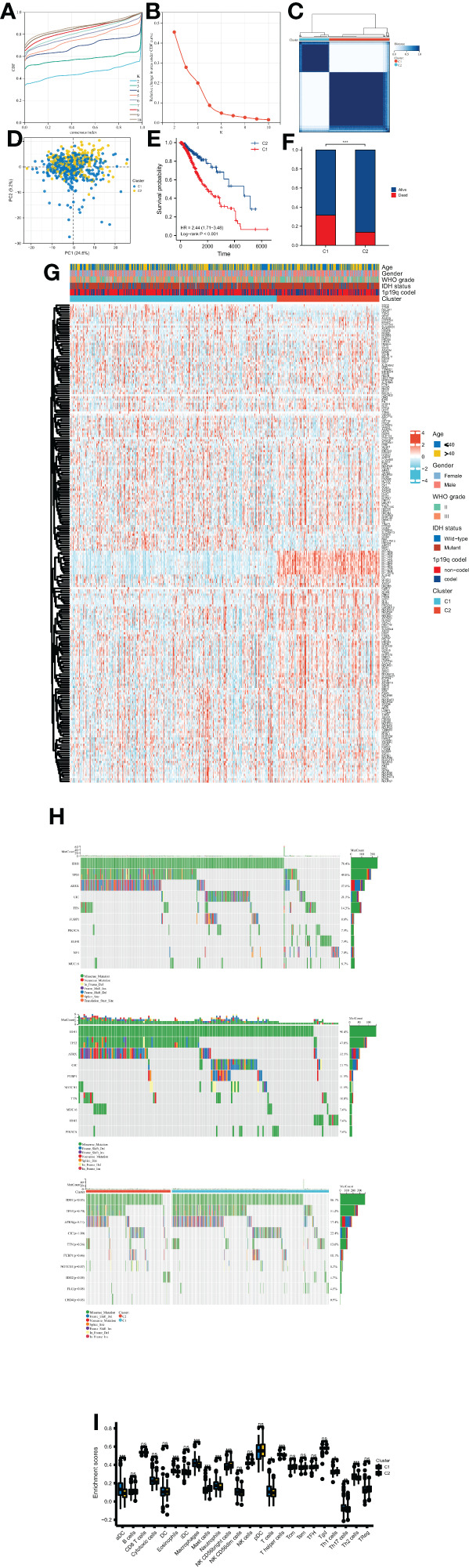
Molecular subtypes of LGG classified by OMRGs. **(A)** CDF curves for consensus scores. **(B)** CDF Delta area curves. **(C)** Two OMRG clusters were defined by consensus clustering analyses. **(D)** Distinction between two OMRG clusters by PCA. **(E)** Kaplan-Meier survival analyses of two OMRG clusters. **(F)** Survival status of patients in different OMRG clusters. **(G)** Heatmap of OMRG expression, clusters, and clinical features. **(H)** Mutation profiles of two OMRG clusters. **(I)** Difference in immune infiltration of two OMRG clusters; ns, not significant; *P<0.05; **P<0.01; ***P<0.001.

After identifying the OMRGs, we performed consensus clustering and classified two clusters based on OMRG expression in LGG patients (**Figures 2A–C**). The principal component analysis (PCA) result showed a separation between the two OMRG clusters (**Figure 2D**). We then compared the survival distribution and found a significantly worse prognosis in cluster 1 (P<0.001, **Figure 2E**). Moreover, we found a significantly higher mortality rate in cluster 1 (P<0.001, **Figure 2F**). We described the clinical features and gene expression in the two OMRG clusters by a heat map (**Figure 2G**). We also analyzed the frequencies of the top 10 mutant genes in the two clusters and found that the mutant frequencies of IDH1, IDH2, and CHD4 were significantly higher in cluster 2, while the mutant frequency of FLG was significantly lower (**Figure 2H**). Furthermore, we found a significant increase in the levels of macrophages, eosinophils, neutrophils, aDC (activated dendritic cells), T cells, T helper cells, Th2 cells, and Th17 cells in cluster 1 (P<0.05 for all, **Figure 2I**).

### Identification of DEGs and risk model construction

**Figure 3:**
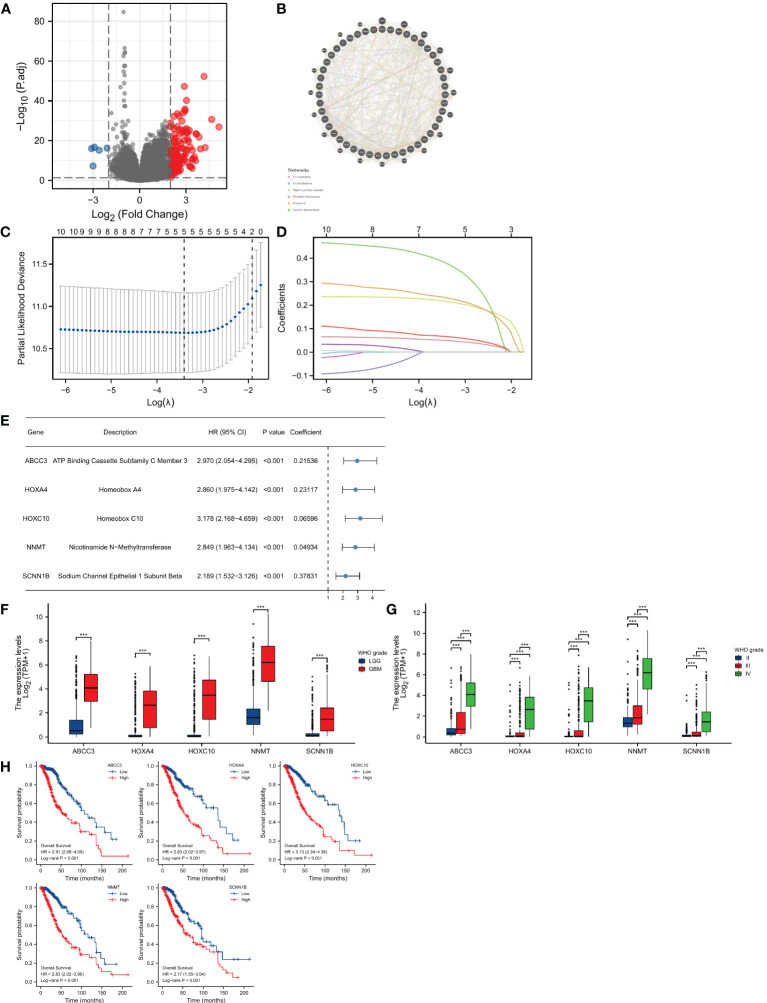
Construction of the prognostic model. **(A)** Identification of DEGs between two OMRG clusters. **(B)** Genetic interaction network of OMRG-related DEGs. **(C)** Cross-validation of the LASSO model parameters. **(D)** Coefficient profiles in LASSO regression model. **(E)** HRs, 95% CIs, and coefficients of signature-genes. **(F)** Difference in expression levels of 5 signature-genes between LGGs and GBMs; ***P<0.001. **(G)** Difference in expression levels of 5 signature-genes among WHO grade II, III, and IV; ***P<0.001. **(H)** Kaplan-Meier curves and log-rank tests of 5 signature-genes.

We identified a total of 132 DEGs, including 127 up-regulated in cluster 1 and 5 up-regulated in cluster 2 (**Figure 3A**). We used GeneMANIA to create genetic interaction networks of these DEGs (**Figure 3B**). To identify prognostic genes within the 132 DEGs of LGG, we performed univariate Cox analyses, which identified 47 genes. We confirmed the gene expression in HPA database, and excluded untested genes and those with poor consistency with protein levels. Eventually, we included 12 genes for LASSO regression, and selected 5 genes to construct the risk model: ABCC3, HOXA4, HOXC10, NNMT, and SCNN1B (**Figures 3C–E**). We found that the levels of these five genes increased significantly with the grade of gliomas (P<0.001 for all, **Figures 3F, G**). Kaplan-Meier analyses revealed significant correlations between high expression levels of these 5 genes and poor prognosis in LGG (P<0.001 for all, **Figure 3H**).

### GSEA analyses

**Figure 4:**
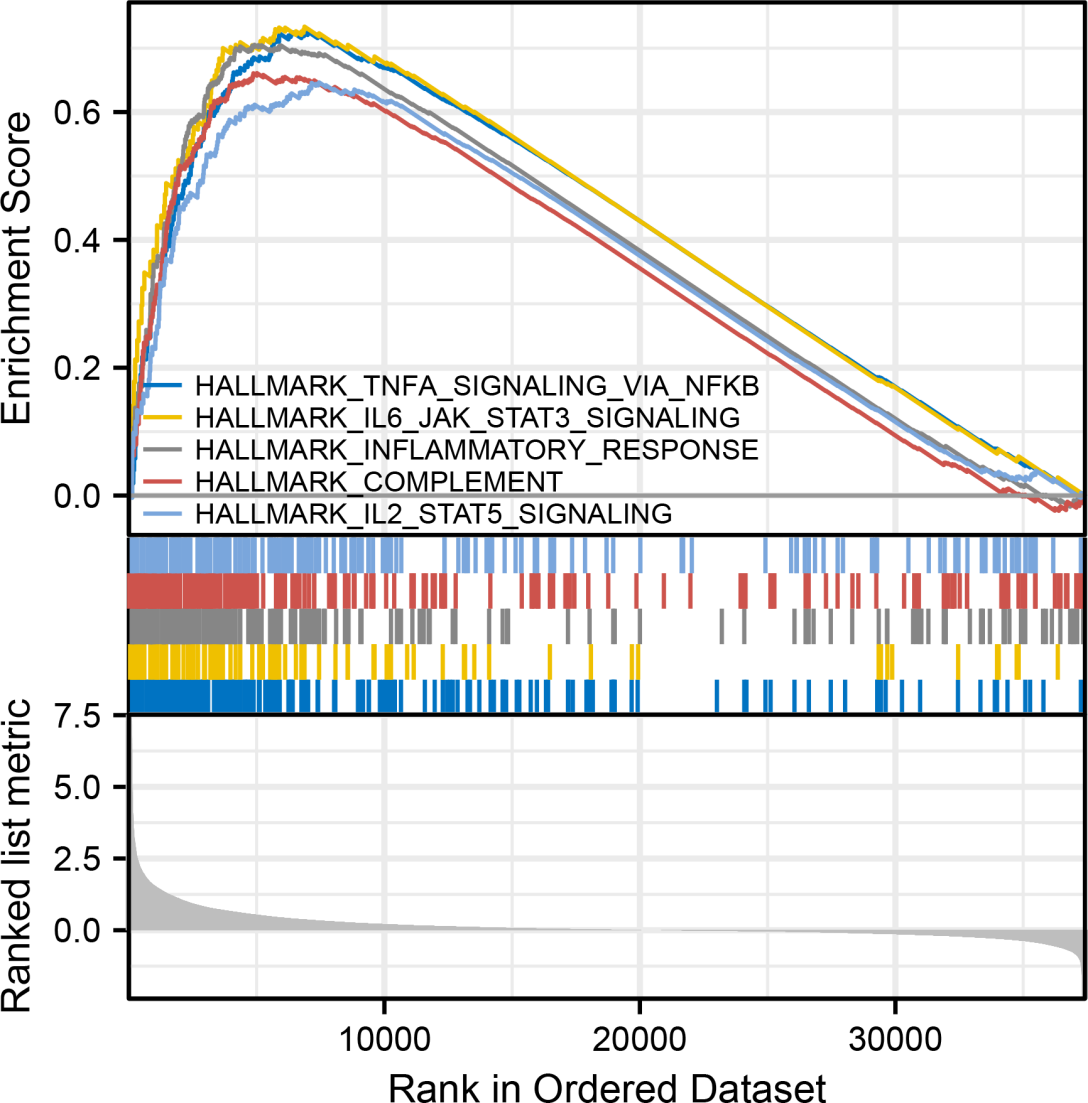
GSEA analyses, including TNFα signaling via NFκB, IL6/JAK/STAT3 signaling, inflammatory response, complement, and IL2/STAT5 signaling.

GSEA has been conducted to identify potential biological function between different risk groups. It showed an enrichment in immune response and signaling in the high-risk group, including TNFα signaling via NFκB, IL6/JAK/STAT3 signaling, inflammatory response, complement, and IL2/STAT5 signaling (**Figure 4**).

### Immune analyses and chemotherapy efficacy

**Figure 5:**
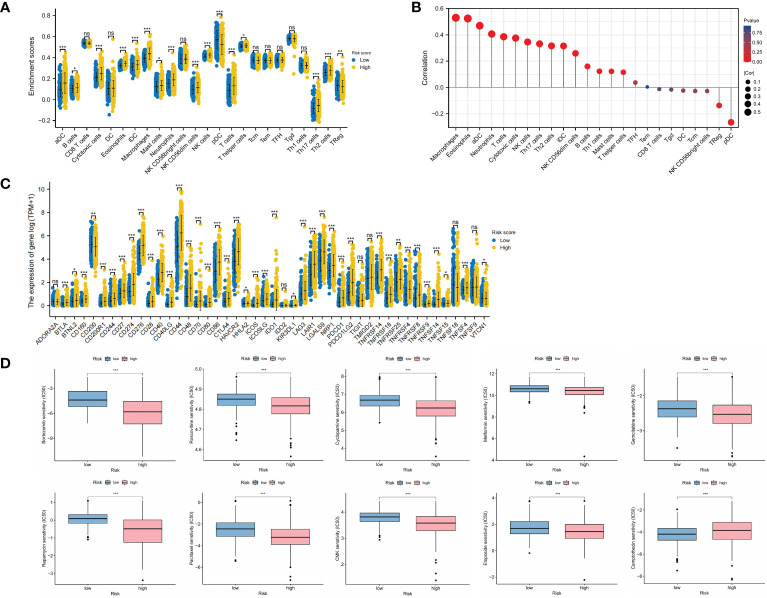
Landscape of immune infiltration, immune checkpoint expression, and drug sensitivity in different risk groups. **(A)** Infiltrating levels of immune cells in low-risk and high-risk groups. **(B)** Correlation between risk score and immune infiltrating levels. **(C)** Expression levels of immune checkpoints in low-risk and high-risk groups. **(D)** Drug sensitivity analyses between low-risk and high-risk groups. *P<0.05; **P<0.01; ***P<0.001.

The comparison of immune infiltration showed significant increase in most immune cells in the high-risk group (**Figure 5A**), and there were significantly positive correlations between OMRG-related risk score and the infiltrating levels of macrophages, eosinophils, aDC, and neutrophils (P<0.001 for all, **Figure 5B**). The levels of immune checkpoints were also compared to predict immunotherapy sensitivity between the two groups and we found significant increase in the levels of most immune checkpoints in the high-risk group (**Figure 5C**). Furthermore, the drug sensitivity between different risk groups was assessed with the IC50 value, and the results revealed that patients in the high-risk group were more sensitive to Bortezomib, Rapamycin, Cyclopamine, Metformin, Gemcitabine, Roscovitine, Paclitaxel, CMK, and Etoposide, while patients in the low-risk group were more sensitive to Camptothecin (P<0.001 for all, **Figure 5D**).

### Risk score distribution and prognostic role

**Figure 6:**
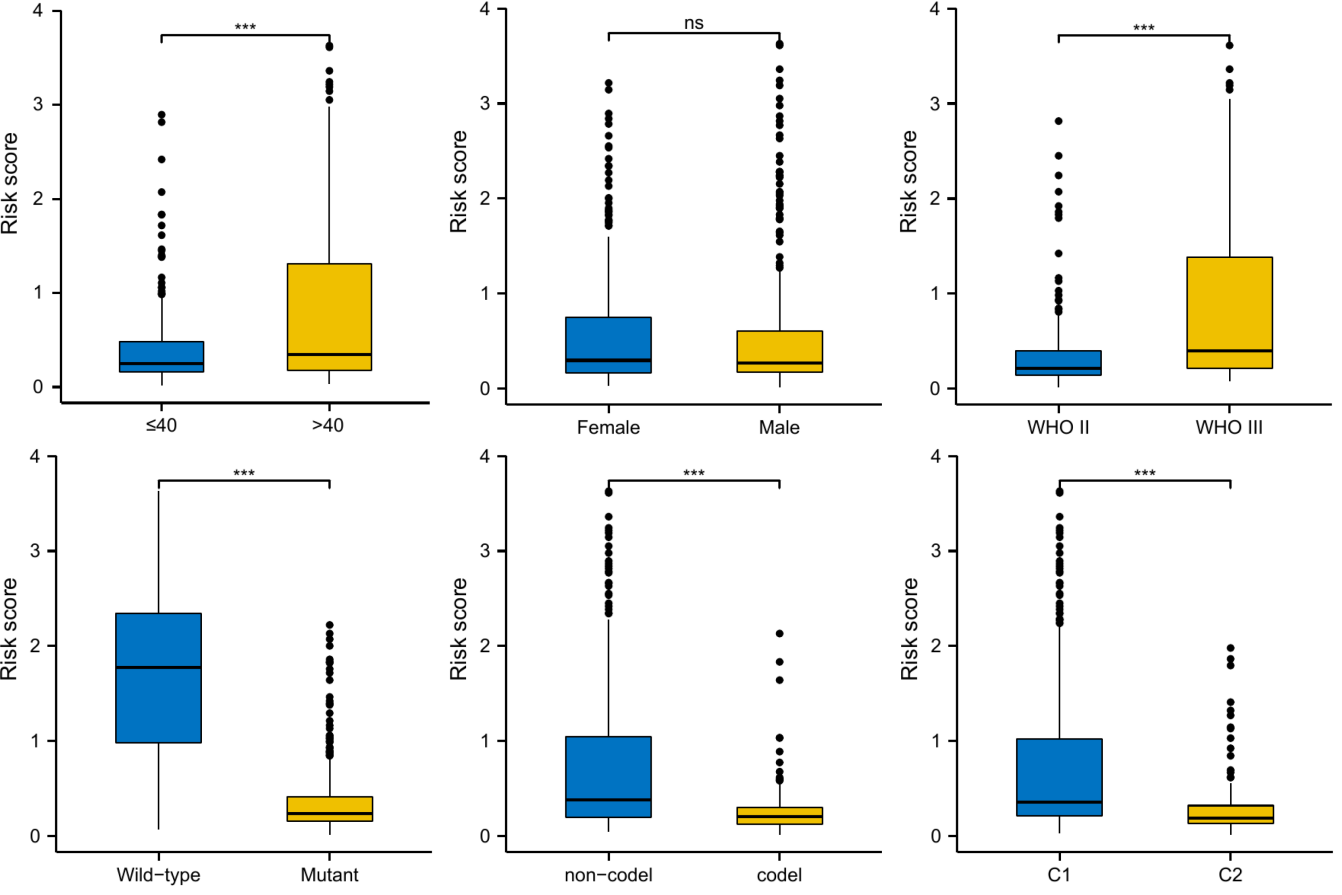
Risk score distribution in different subgroups of LGG, including age, gender, WHO grade, IDH mutation, 1p/19q co-deletion, and OMRG clusters; ns, not significant; ***P<0.001.

**Figure 7:**
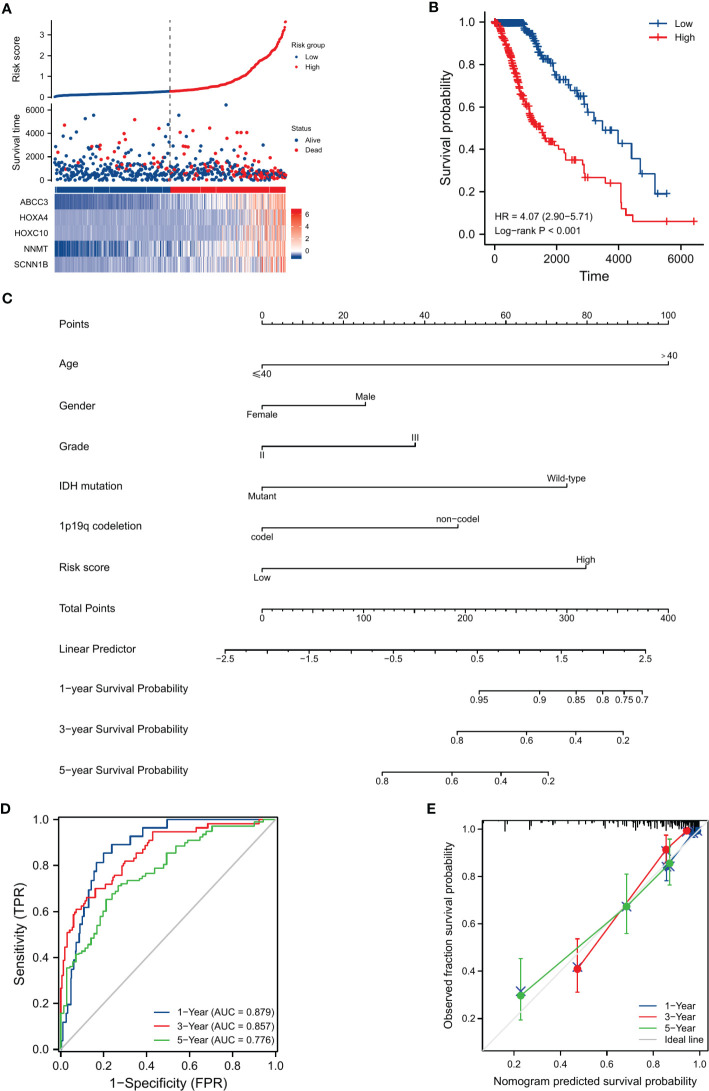
Evaluation of the prognostic efficiency of risk score in the OS of LGG patients. **(A)** The distribution of risk score, survival status, and signature-gene expression in low-risk and high-risk groups. **(B)** Kaplan-Meier analysis for survival probability estimation in different risk groups. **(C)** Nomogram for 1-year, 3-year, and 5-year OS rate prediction of LGG patients. **(D)** Time-dependent ROC curves. **(E)** Calibration plots of the nomogram model.

We compared the distribution of risk scores across different clinical subgroups (**Figure 6**). And we compared the survival distribution and levels of the five signature-genes (**Figure 7A**). The Kaplan-Meier curve indicated that the prognosis of the high-risk group was significantly worse (P<0.001, **Figure 7B**). Cox analyses identified the prognostic role of OMRGs-related risk score in LGG (HR=2.665, 95%CI=1.626-4.369, P<0.001, **Table 1**). Then we used the same variables to create a nomogram predicting the probabilities of OS rates (**Figure 7C**). The AUCs of time-dependent ROC curves have been shown (1-year=0.879, 3-year=0.857, 5-year=0.776, **Figure 7D**). Calibration plots showed satisfactory consistency between the nomogram and the ideal model (**Figure 7E**).

**Table 1:** Univariate and multivariate Cox analyses of LGG patients from TCGA dataset.

Characteristics	Univariate analysis		Multivariate analysis	
	Hazard ratio (95% CI)	P value	Hazard ratio (95% CI)	P value
Age				
≤40	Reference		Reference	
>40	2.889 (2.009-4.155)	<0.001*	3.432 (2.202-5.349)	<0.001*
Gender				
Female	Reference			
Male	1.124 (0.800-1.580)	0.499		
WHO grade				
II	Reference		Reference	
III	3.059 (2.046-4.573)	<0.001*	1.598 (1.021-2.502)	0.040*
IDH status				
Wild-type	Reference		Reference	
Mutant	0.186 (0.130-0.265)	<0.001*	0.440 (0.276-0.703)	<0.001*
19q codeletion				
Non-codel	Reference		Reference	
Codel	0.401 (0.256-0.629)	<0.001*	0.551 (0.328-0.927)	0.025*
Risk score				
Low	Reference		Reference	
High	4.144 (2.784-6.168)	<0.001*	2.665 (1.626-4.369)	<0.001*

*P<0.05, significant difference.

### Validation of prognostic role

**Figure 8:**
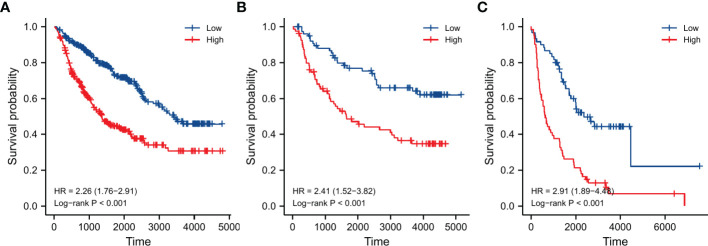
Validation of survival analyses in different external datasets. **(A)** CGGA sequencing dataset. **(B)** CGGA microarray dataset. **(C)** REMBRANDT dataset.

A total of 961 LGG samples from three external databases were used for validation. Cox regression in CGGA-seq dataset verified the prognostic role of OMRG-related risk score (HR=1.489, 95%CI=1.102-2.012, P=0.010, **Table 2**). Furthermore, Kaplan-Meier analyses by the CGGA-sequencing set, CGGA-microarray set, and REMBRANDT cohort showed a significant association between high-risk score and poor prognosis in LGG (P<0.001 for all, **Figures 8A–C**).

**Table 2:** Univariate and multivariate Cox analyses of LGG patients from CGGA sequencing dataset.

Characteristics	Univariate analysis		Multivariate analysis	
	Hazard ratio (95% CI)	P value	Hazard ratio (95% CI)	P value
Age				
≤40	Reference			
>40	1.249 (0.973-1.604)	0.081		
Gender				
Female	Reference			
Male	0.840 (0.654-1.080)	0.174		
WHO grade				
II	Reference		Reference	
III	2.808 (2.141-3.682)	<0.001*	2.906 (2.176-3.880)	<0.001*
IDH status				
Wild-type	Reference		Reference	
Mutant	0.428 (0.327-0.561)	<0.001*	0.738 (0.548-0.994)	0.046*
1p/19q codeletion				
Non-codel	Reference		Reference	
Codel	0.256 (0.179-0.364)	<0.001*	0.341 (0.231-0.505)	<0.001*
Risk score				
Low	Reference		Reference	
High	2.236 (1.725-2.898)	<0.001*	1.489 (1.102-2.012)	0.010*

*P<0.05, significant difference.

### Validation of gene expression

**Figure 9:**
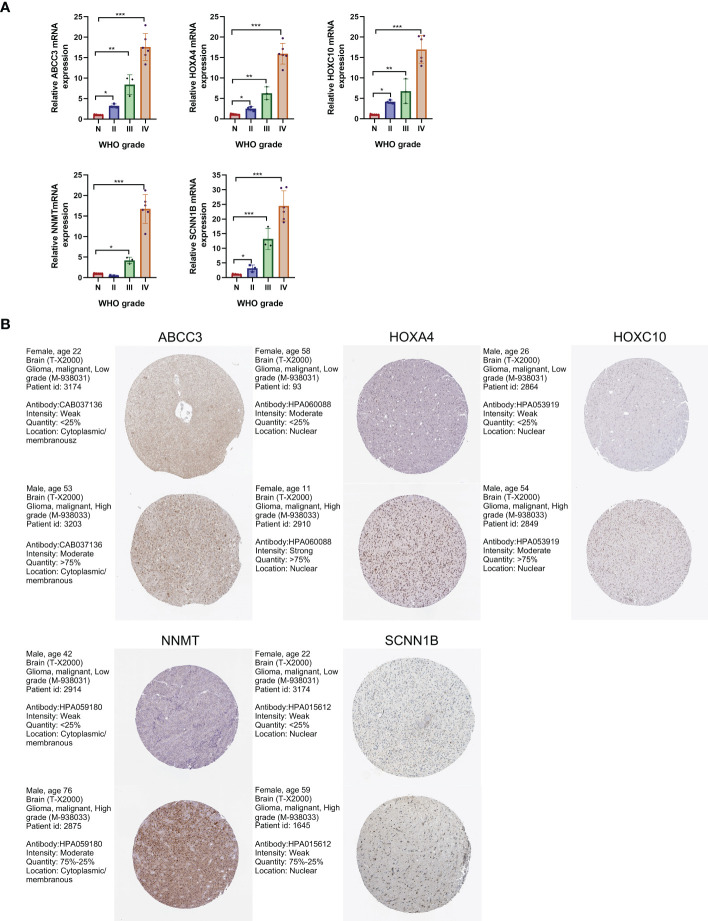
Validation of gene expression levels by qRT-PCR and IHC analyses. **(A)** Expression levels of the 5 signature-genes in glioma tissues by qRT-PCR analyses. **(B)** Protein expression levels of the 5 signature-genes in glioma tissues by IHC results. *P<0.05; **P<0.01; ***P<0.001.

To verify the expression of the five signature-genes, we conducted qRT-PCR analyses on glioma tissues with different grades. The expression levels of ABCC3, HOXA4, HOXC10, NNMT, and SCNN1B significantly increased with the elevation in WHO grades (**Figure 9A**). To further validate our findings, we analyzed the protein expression levels of these genes using IHC results from the HPA database. The results showed that the protein expression levels of these genes were up-regulated with the malignant degree of gliomas, which was consistent with the trend observed in the gene expression (**Figure 9B**).

### Knockdown of SCNN1B decreased the migration *in vitro*

**Figure 10:**
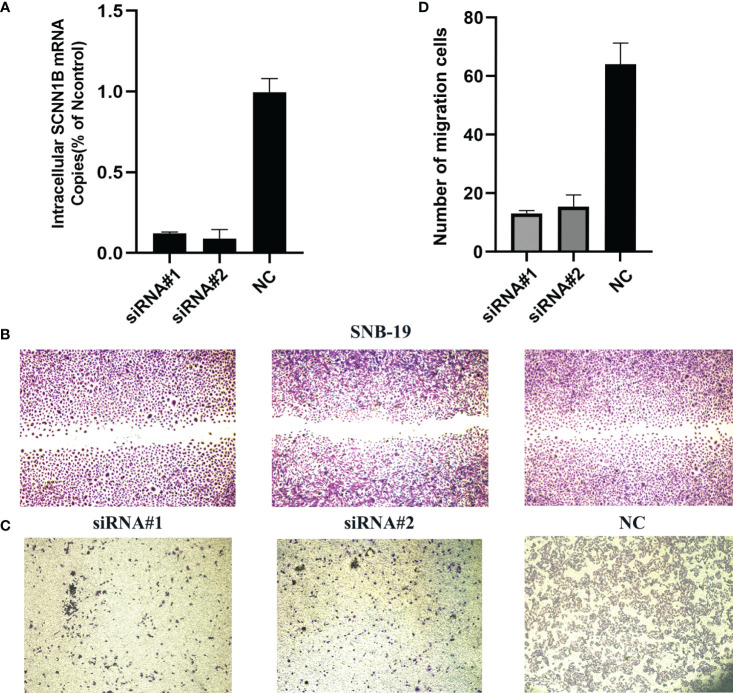
Biological function of SCNN1B in SNB-19 cell line. **(A)** Transfection efficiency of siRNA. **(B)** Wound healing assays. **(C, D)** Transwell assays.

To further determine the biological function of SCNN1B, we utilized siRNAs to knockdown SCNN1B in SNB-19 glioma cell line (**Figure 10A**). The results demonstrated a significant decrease in the ability of wound healing in SNB-19 cells after knockdown of SCNN1B (**Figure 10B**). Additionally, the transwell assay revealed a consistent decrease in migration ability in SNB-19 cells after SCNN1B knockdown (**Figures 10C, D**; #1, P<0.001; #2, P<0.001), indicating the crucial role of SCNN1B in gliomas.

## Discussion

As the most prevalent type of malignant intracranial tumor, glioma shows an infiltrative growth pattern (30). Despite the development of effective therapeutic options, the OS rates of LGG patients exhibit a considerable interindividual variability due to the high incidence of local recurrence, malignant progression (31, 32). In recent years, molecular biomarkers have been found to have prognostic significance in gliomas. However, accurately predicting the prognosis of LGG patients has remained challenging. Mitochondria play a key role in cellular energy production and calcium homeostasis. The mtDNA mutations, respiratory chain malfunction, and oxidative phosphorylation disruption caused by ROS accumulation could lead to mitochondrial dysfunction, which in turn exacerbated oxidative stress (33). Previous studies have confirmed that mitochondrial dysfunction resulted in metabolic reprogramming, cell metabolic pathway alternation, and redox balance damage, which are closely associated with genetic instability and the occurrence of tumors. Mitochondrial dysfunction-related metabolic reprogramming is a hallmark of tumor cells to meet the energy and biosynthetic demands required for uncontrolled proliferation and survival (34). Mitochondrial dysfunction can induce metabolic reprogramming in tumor cells, including Warburg effect, glutaminolysis, and fatty acid oxidation. The Warburg effect is a well-known phenomenon in which tumor cells preferentially use glycolysis for energy production, even in the presence of oxygen (35). Glutaminolysis is an important metabolic pathway in tumor cells that utilizes glutamine as a carbon source for the synthesis of nucleotides, amino acids, and lipids (36). Fatty acid oxidation is also upregulated in tumor cells to meet the increased demand for energy and biosynthesis (37). Mitochondrial dysfunction can alter cell metabolic pathways, leading to the accumulation of oncometabolites, such as 2-hydroxyglutarate (2-HG), which can promote oncogenic signaling and epigenetic alterations (38). It can also lead to changes in redox imbalance. As a byproduct of oxidative phosphorylation, ROS can cause oxidative damage to cellular components, including DNA, proteins, and lipids. Increased ROS levels could contribute to tumorigenesis by promoting genomic instability, activating oncogenic signaling pathways, and suppressing immune surveillance (39). Mitochondrial dysfunction-related alterations in metabolic reprogramming, cell metabolic pathways and redox balance are closely associated with the occurrence and progression of tumors. Furthermore, glycolysis and lactic acid synthesis caused by mitochondrial dysfunction and chronic inflammation caused by oxidative stress might contribute to immune infiltration in the tumor immune microenvironment (TIM) (40). Therefore, it is of great significance to identify different molecular subtypes and explore potential glioma prognostic biomarkers in LGG patients based on OMRGs.

In this study, we first obtained the list of OMRGs as the intersection of ORGs and MRGs. The potential functions of OMRGs were confirmed by functional enrichment analyses. We then performed consensus clustering analysis to identify different molecular subtypes. We identified two OMRG clusters and compared the survival probabilities between the two clusters. We observed that the outcome of patients in cluster 1 was significantly worse, and the mortality in cluster 1 was significantly higher. These were consistent with the results of mutant profiles. It showed a significantly higher prevalence of IDH1 and IDH2 gene alterations in cluster 2. According to previous studies, IDH mutant gliomas exhibited less aggressive biological behaviors and showed a better prognosis and chemotherapy response, independent of histopathological grades (41). The increased level of infiltrating immune cells in cluster 1 indicated an immunosuppressive and chronic inflammatory microenvironment in these samples.

We identified DEGs and confirmed the prognostic role of these genes. Using LASSO regression, five genes were selected for risk model construction, including ABCC3, HOXA4, HOXC10, NNMT, and SCNN1B. These five genes were significantly increased with the grade of glioma and the high expression levels were significantly associated with the poor prognosis in LGG. ABCC3 is a member of the ATP-binding cassette transporter superfamily, it is strongly associated with tumor drug resistance, leading to chemotherapy failure (42). ABCC3 has been found highly expressed in different types of tumors, and high ABCC3 expression significantly predicted a shorter OS in glioma (43). This has been considered to be associated with the impaired temozolomide reaction. A recent study identified two ABCC3-targeting nanobodies as novel candidates for immunotargeting applications in GBM (44). HOXA4 belongs to the Homeobox gene family. HOX genes encode transcription factors and control the process of cell differentiation (45). HOXA4 overexpression promoted self-renewal and overpopulation of colon cancer stem cells (46). A recent study found that HOXA4 knockdown could block cell cycle pathway and inhibit the proliferation, invasion, and chemotherapy resistance in gliomas (47). A series of studies have explored the function of HOXC10 in gliomas. Up-regulation of HOXC10 promoted an aggressive phenotype in glioma and induced the expression of genes involved in tumor immunosuppression (48). Additionally, HOXC10 up-regulated the expression of VEGFA, enhancing the capacity of glioma angiogenesis, which made it a potential therapeutic target for antiangiogenic therapy (49). Interestingly, a recent study revealed that upregulation of HOXC10 in ovarian cancer could promote ABCC3 expression by transcriptional upregulation of β-catenin, resulting in carboplatin resistance (50). NNMT is a member of N-methyltransferase family and contributed to tumorigenesis. NNMT overexpression was associated with the invasion of glioma cells and cellular methylation reorganization, leading to the down-regulation of downstream protein GAP43 (51). In contrast, NNMT silencing could activate tumor suppressor PP2A and inactivate oncogenic STKs, thereby inhibiting glioma-forming ability and enhancing radiation sensitivity (52). SCNN1B is located on chromosome 16p12-p13. Previous studies have explored the role of SCNN1B in gastrointestinal tumors. SCNN1B interacted with GRP78 and induced its degradation, which led to Caspase-dependent apoptosis and ultimately inhibited cell growth and migration in gastric cancer (53). Furthermore, SCNN1B could inhibit the growth of colorectal cancer by impairing the activation of c-Raf and suppressing MAPK signaling (54).

We performed risk score calculations and stratified LGG patients into different risk groups. The GSEA analyses revealed a significant difference in immune response and signaling between these two groups. We then analyzed the immune infiltration patterns and the expression levels of immune checkpoints in both groups. We found significant correlations between the risk score and the infiltrating levels of macrophages, eosinophils, aDC, neutrophils, and T cells. TIM has always been the focus of research, which played a crucial role in tumorigenesis, development, and chemotherapy resistance. Glioma-related macrophages, DCs, and neutrophils contributed to glioma microenvironment, which regulated and inhibited the tumor immune response (55). In tumor microenvironment (TME), the balance of macrophages shifted from anti-tumor activated M1 to tumor-promoting activated M2 (56). The increased levels of M2 macrophages produced numerous cytokines, growth factors, and interleukins, which facilitated the immunological tolerance and promoted a tumor-permissive microenvironment in glioma. As professional antigen-presenting cells, DCs regulated the immune response activation of T cells (57). The elevated levels of aDCs and T cells indicated the chronic inflammation in TME that promoted tumor progression. Eosinophils could produce matrix metalloproteinases (MMP) and growth factors, which interacted with epidermal growth factor receptors overexpressed in glioma and promoted tumor progression (58). Neutrophils have been shown to possess both protumorigenic and antitumorigenic properties in TME (59). Neutrophils could secrete immunosuppressive mediators, including ROS, chemokines, and MMP-9, which contributed to a pro-tumor microenvironment. Moreover, the vascular endothelial growth factor (VEGF) produced by neutrophils in TME could promote angiogenesis and tumor progression (60). The immunosuppressive microenvironment and chronic inflammation might be the major reason for poor outcome of LGG patients with high OMRG-related risk score. The high levels of immune checkpoint expression in the high-risk group suggested the potential efficacy of immunotherapy in these patients. We performed drug sensitivity analyses and found that the high-risk patients were more sensitive to most chemotherapeutic agents, providing a novel perspective for chemotherapy treatment.

Subgroup OMRg-related risk score comparisons suggested a potential correlation between poor prognosis and high-risk score in LGG patients. The subsequent survival analyses and Cox regression analyses identified the prognostic role of OMRG-related risk score. To predict the survival probability, we constructed a nomogram model with the same clinical features as Cox regression. It showed a favorable predictive efficiency of the model. We confirmed the prognostic role of OMRG-related risk score with a total of 1490 LGG samples from four different datasets. The findings were consistent and stable. The results of qRT-PCR and IHC staining confirmed the expression of the five selected genes. Previous studies have demonstrated the function of ABCC3, HOXA4, HOXC10, and NNMT in gliomas. However, the function of SCNN1B has not been studied. The results of *in vitro* experiments showed that high SCNN1B expression promoted the migration of glioma cells. However, there were still several limitations in our study. This study was based on retrospective analyses and the prognostic role of the signature should be verified in multi-center large-sample prospective cohorts. Additionally, the signature gene-related signaling pathways and regulatory mechanisms remained to be further studied.

## Conclusion

In this study, we have identified two molecular subtypes of LGG based on OMRGs, providing insights into the potential combined effect of mitochondrial dysfunction and oxidative stress in LGG. Moreover, we have established a novel OMRG-related gene signature that could be utilized for predicting outcomes, immune status, and therapeutic efficiency in LGG patients.

## Data availability 

The original contributions presented in the study are included in the article/**Supplementary Material**. Further inquiries can be directed to the corresponding authors.

## Ethics statement

The studies involving human participants were reviewed and approved by Ethics Committee in Beijing Tiantan Hospital, Capital Medical University. The patients/participants provided their written informed consent to participate in this study.

## Author contributions

JL illustrated the results and completed the manuscript. XC performed the experiment operation. WW and CT performed the consensus clustering analyses, immune analyses, and drug sensitivity analyses. QH and YD obtained the data from public databases and conducted the statistical analyses. SW and JW revised and polished the manuscript. JZ and WW conceived and designed this study. All authors contributed to the article and approved the submitted version.
