# Supplementary material for: Identification of heterogeneous subtypes and a prognostic model for gliomas based on mitochondrial dysfunction and oxidative stress-related genes
Source: Front Immunol. 2023 Jun 2;14:1183475. doi: 10.3389/fimmu.2023.1183475 (PMC10272431; doi:10.3389/fimmu.2023.1183475)
Supplement: Supplementary file 1 [file Table_1.docx]

| Gene | Forward Primer (5' to 3') | Reverse Primer (5' to 3') |
| --- | --- | --- |
| ABCC3 | GGTCCCTAAAGGAAGAGGACAGA | ATTTTTCCCAGGTGCTGCTGAAG |
| HOXA4 | ATAACGGAGGGGAGCCTAAG | GCTCAGACAAACAGAGCGTG |
| HOXC10 | CTCGGATAACGAAGCGAAAG | CGCTCTCGCGTCAAATACAT |
| NNMT | GTTTGGTTCTAGGCACTCTGCAG | AGAGCCGATGTCAATCAGCAGG |
| SCNN1B | CCCTGATCGCATAATCCTAGC | ATGCCCCAGTTGAAGATGTAG |

Supplementary Table S1. Primer sequences for qRT-PCR.
